# The EC_90_ of remifentanil for blunting cardiovascular responses to head fixation for neurosurgery under total intravenous anesthesia with propofol and remifentanil based on bispectral index monitoring: estimation with the biased coin up-and-down sequential method

**DOI:** 10.1186/s12871-017-0426-z

**Published:** 2017-10-10

**Authors:** Jung-Man Lee, Jae-Hyon Bahk, Young-Jin Lim, Jiwon Lee, Leerang Lim

**Affiliations:** 1 Department of Anesthesiology and Pain Medicine, Seoul Metropolitan Government Seoul National University Boramae Medical Center, 20, Boramae-ro 5-gil, Dongjak-gu, Seoul 07061 Republic of Korea; 2 Department of Anesthesiology and Pain Medicine, Seoul National University Hospital, Seoul National University College of Medicine, 101, Daehak-ro, Jongno-gu, Seoul 03080 Republic of Korea; 3 Department of Anesthesiology and Pain Medicine, Keimyung University Dongsan Medical Center, Keimyung University College of Medicine, 56, Dalseong-ro, Daegu, 41931 Republic of Korea; 4 Department of Anesthesiology and Pain Medicine, Seoul National University Hospital, 101, Daehak-ro, Jongno-gu, Seoul 03080 Republic of Korea

**Keywords:** Head fixation, Neurosurgery, Remifentanil

## Abstract

**Background:**

Head fixation can induce hemodynamic instability. Remifentanil is commonly used with propofol for total intravenous anesthesia (TIVA) during neurosurgery. This study investigated the 90% effective concentration (EC_90_) of remifentanil for blunting of cardiovascular responses to head fixation during neurosurgery via bispectral index (BIS) monitoring.

**Methods:**

Fifty patients undergoing neurosurgery requiring head fixation were enrolled. This study was performed using the biased coin up-and-down design sequential method (BCD). After tracheal intubation, the effect-site target concentration (Ce) of remifentanil was adjusted to achieve hemodynamic stability and reset to the level preoperatively assigned to each patient, according to the BCD method, approximately 10 min before head fixation. Baseline hemodynamic values were recorded before head fixation. An ineffective response was defined as a case with a > 20% increase in hemodynamic values from baseline. Otherwise, the response was determined to be effective. The EC_90_ of remifentanil was calculated as a modified isotonic estimator.

**Results:**

Forty-three patients completed this study. The EC_90_ of remifentanil for blunting cardiovascular responses to head fixation was estimated to be 6.48 ng/mL (95% CI, 5.94–6.83 ng/mL).

**Conclusions:**

Adjustment of the Ce of remifentanil to approximately 6.5 ng/mL before head fixation could prevent noxious cardiovascular responses in 90% of neurosurgical ASA I-II patients aged 20 to 65 years old during propofol target-controlled infusion titrated to maintain BIS between 40 and 50.

**Trial registration:**

ClinicalTrials.gov Identifier NCT01489137, retrospectively registered 5 December 2011.

## Background

A head holder is commonly used to stabilize the head during neurosurgery. Skull pin insertion, head pinning, and pin fixation are synonyms for head fixation and indicate the application of a head holder. Use of a head holder is of paramount importance during stereotactic neurosurgery. However, anesthesiologists may encounter hemodynamic changes that require pharmacological intervention due to a noxious stimulus resulting from head fixation. Acute arterial hypertension can lead to intracranial hemorrhage [1, 2]. Therefore, various strategies have been used to reduce the degree of hemodynamic changes induced by head fixation, resulting in various recommendations [3–7].

Recently, total intravenous anesthesia (TIVA) has become a widely used method for general anesthesia. Among various combinations of intravenous anesthetics, continuous propofol infusion and opioid supplementation is a widely used combination. TIVA is considered a standard method of general anesthesia, especially for evoked potential monitoring [8]. Due to its pharmacodynamic and pharmacokinetic characteristics, such as a very short context-sensitive half-time and minimal effects on cardiovascular system, remifentanil is a commonly used opioid in conjunction with propofol for TIVA.

This study was designed to estimate the 90% effective concentration (EC_90_) of remifentanil for blunting cardiovascular responses to head fixation during neurosurgery under TIVA with bispectral index (BIS) monitoring.

## Methods

This study was approved by the Institutional Review Board of the Seoul National University Hospital. Written informed consent was obtained from all patients. The trial was registered at www.clinicaltrials.gov (NCT01489137). American Society of Anesthesiologists physical status I-II patients were enrolled in this study. The patients were 20–65 years old and were scheduled to undergo elective neurosurgery requiring head fixation. Patients who were obese (body mass index >30.0) or severely underweight (body mass index <16.0), had hypertension, cardiac disease, pulmonary disease, or renal disease, used current medication affecting the cardiovascular system, or were addicted to substances or alcohol were excluded from this study. Furthermore, patients who were administered any drugs that affect the cardiovascular system, such as ephedrine, during the period from the induction of anesthesia to head fixation were excluded.

### Anesthesia

Prior to the surgery, patients fasted for 8 h and received no premedication. A standard monitoring and anesthetic technique was applied to all patients in the operating room. All patients received TIVA with propofol and remifentanil using a target-controlled infusion (TCI) system under BIS monitoring with the BIS VISTA™ system (Aspect Medical Systems, Newton, MA, USA). The Schnider and Minto models were selected as the pharmacokinetic models for propofol and remifentanil, respectively [9, 10]. The TCI mode was set to the effect-site control using an Orchestra™ device (Fresenius Vial, France).

The same effect-site target concentrations (Ce) of propofol (4 μg/mL) and remifentanil (4 ng/mL) were given to all patients for anesthetic induction. After induction of anesthesia, the lungs of patients were ventilated via manual bagging, and 0.8 mg/kg rocuronium was administered. After muscle relaxation, an anesthesiologist inserted a 20-G catheter into the radial artery to monitor continuous arterial blood pressure during the operation. Then, tracheal intubation was performed. Infusion of 0.9% normal saline (10 mL/kg) was performed during the trial to compensate for dehydration from overnight fasting, which was completed before recording baseline hemodynamics.

### Study design and setting

At approximately 10 min before head fixation, the propofol Ce was adjusted to maintain the BIS at approximately early 40s, but over than 40. During the remainder of the operation, the propofol Ce was adjusted to maintain the BIS at between 40 and 50. After endotracheal intubation, the remifentanil Ce was adjusted to stabilize the hemodynamic responses. The remifentanil Ce was reset to the preoperatively assigned level at least 10 min before the initiation of head fixation. This was performed by the same anesthesiologist in each case: the anesthesiologist opened an envelope containing a card stating the remifentanil Ce assigned to the patient and then adjusted the Ce to the assigned level by modulating the TCI pump for the patient. A second, standing anesthesiologist managed the patient for the whole operation and recorded the vital signs during head fixation. The standing anesthesiologist was blinded to the remifentanil Ce by covering the screen of the Orchestra™ device with a sheet of paper during the study. Before the study, the standing anesthesiologist was ordered to only administer vasoactive drugs such as ephedrine in cases in which the mean arterial blood pressure (MAP) decreased to <55 mmHg before head fixation. The MAP and heart rate (HR) were recorded by a standing anesthesiologist at 1 and 2 min before head fixation, and the means of these readings were calculated and used as the baseline values (MAP_BL_ and HR_BL_). Neurosurgeons performed head fixation without any local infiltration of anesthetics at the pin site or use of a scalp nerve block. The MAP and HR were monitored and recorded during head fixation and the immediate post-fixation period.

**Fig. 1:**
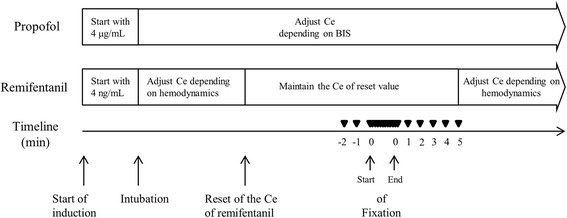
Timeline of the study. ▼, recording point of hemodynamic parameters

The peak values during head fixation were recorded to determine their primary end-points, which were defined as the percentage increases compared to the MAP_BL_ and HR_BL_ ([peak value – baseline]/baseline × 100%). After completion of head fixation, the MAP and HR were observed for 5 min, and the values were recorded each minute. Figure 1 shows the timeline of the study.

A vasoactive drug or β-agonist was prepared for administration if the MAP decreased to <55 mmHg or the HR decreased to <45/min. Subjects who were administered these drugs before head fixation were excluded from the study. We were also prepared to inject appropriate drugs if the MAP and HR did not return to within 120% of baseline values within 3 min after head fixation.

### Statistical analyses

The EC_90_ of remifentanil that attenuated the cardiovascular responses to head fixation was estimated using the biased coin up-and-down design sequential method (BCD) [11]. The remifentanil Ce for the first subject was set to 5.5 ng/mL based on our past clinical experience. The remifentanil Ce for the next patient was determined by the responses of the previous subject who had completed the trial; a Ce difference of 0.5 ng/mL was chosen as our step size. In case of failure to attenuate the hemodynamic responses to head fixation, (> 20% increase in MAP_BL_ or HR_BL_), the following subject would receive a higher Ce at an increment of 0.5 ng/mL. If the change was within a 20% increase for both the MAP_BL_ and HR_BL_ (thus, the remifentanil Ce was effective), the remifentanil Ce for the next subject was randomly assigned with a probability of 0.89 (8/9) of a Ce decrement of 0.5 ng/mL from the prior subject’s Ce or a probability of 0.11 (1/9) of the same Ce as the prior subject. If a subject was excluded for any reason, the subsequent subject was given the Ce assigned to the excluded subject.

A sample size of at least 40 was determined according to a statistical reference [12]. The EC_90_ was estimated by calculating a modified isotonic estimator (MIE) [12]. The R 2.14.1 program (R foundation for Statistical Computing, Vienna, Austria) was used for this calculation. The 95% confidence interval (CI) was obtained using a parametric bootstrap routine and calculated by a bias-corrected percentile method [11].

## Results

Fifty patients were enrolled in the study. Seven patients were excluded due to the use of ephedrine to treat hypotension prior to head fixation (*n* = 4), inadequate steady-state time (< 10 min) from reset of the remifentanil Ce to head fixation (*n* = 2), and topical infiltration of 1% lidocaine with epinephrine (1:200,000) for nasal mucosa vasoconstriction prior to head fixation in a tumorectomy with a trans-sphenoidal approach (*n* = 1).

**Fig. 2:**
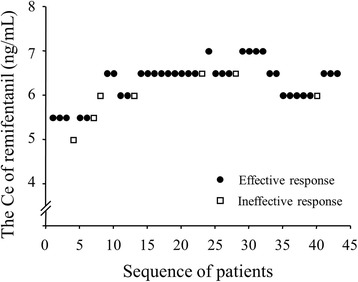
Remifentanil Ce assignment and patient responses. The remifentanil Ce for the first subject was 5.5 ng/mL. The Ce assigned to sequential subjects was determined by the biased coin design up-and-down sequential method. Ce, effect-site target concentration

A total of 43 patients completed this study, and their data were analyzed. The remifentanil Ce ranged from 5.0 to 7.0 ng/mL according to the BCD method (Fig. 2).

The patients’ clinical characteristics are listed in Table 1. Among the 43 subjects who completed the study, seven experienced hemodynamic changes characterized by a > 20% increase in their MAP_BL_ or HR_BL_ values. Thus, the remifentanil Ce used in these subjects was not sufficient to alleviate their hemodynamic responses to head fixation. Although these were recorded as failures, the severity of the change was not sufficient to require antihypertensive drugs. The hemodynamic changes in the remaining subjects were characterized by ≤20% increases from baseline values, and these were considered effective cases. In all cases, the MAP and HR returned to within 120% of the baseline value within 2 and 1 min, respectively.

**Table 1:** Clinical characteristics of the 43 subjects who completed the study

Variables	Mean ± SD
Males / females (n)	20^a^ / 23^a^
Age (y)	46.6 ± 11.0
Weight (kg)	64.1 ± 11.2
Height (cm)	163.5 ± 9.1
BMI (kg/m^2^)	23.9 ± 3.1
Ce of Propofol during fixation (μg/mL)	2.9 ± 0.7
BIS prior to fixation	41.2 ± 2.0
Baseline MAP (mmHg)	77.4 ± 11.7
Baseline HR (beats/min)	61.7 ± 9.8

*BMI* body mass index. Ce, effect-site target concentration. The results are presented as the mean ± SD or numbers^a^

The BIS of 42 of the 43 subjects increased. The change in the BIS among all 43 subjects was 6.6 ± 3.0 (mean ± SD). The BIS decreased by only 1 in one subject.

**Fig. 3:**
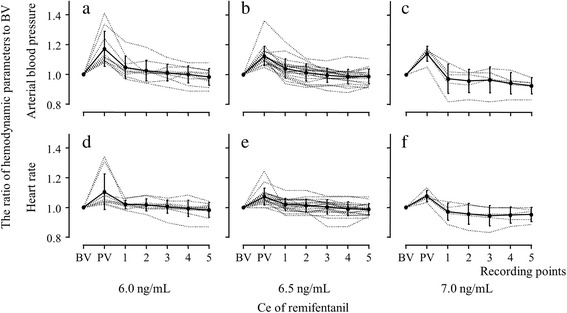
Changes in the mean arterial blood pressure and heart rate of subjects with remifentanil Ce values of 6.0 (*n* = 10), 6.5 (*n* = 21) and 7.0 (*n* = 5) ng/mL. The Y axis in all figures [(**a**) to (**f**)] represents the ratio of hemodynamic parameters to baseline values. The X axis represents the time-points at which hemodynamic parameters were recorded. **a**, **b**, and **c** show the arterial blood pressure ratio compared to baseline values in patients assigned to a remifentanil Ce of 6.0, 6.5 and 7.0 ng/mL, respectively. **d**, **e**, and **f** show the heart rate ratio compared to baseline values in patients assigned to a remifentanil Ce of 6.0, 6.5 and 7.0 ng/mL, respectively. BV indicates the baseline values of hemodynamic parameters; PV indicates peak value of hemodynamic parameters immediately after head fixation; Ce is the effect-site target concentration; the numbers 1, 2, 3, 4 and 5 on the X-axis represent the time (min) after fixation

The calculated EC_90_ of remifentanil was 6.48 ng/mL (95% CI, 5.96–6.83 ng/mL). Figure 3 shows changes in the MAP and HR in subjects with remifentanil Ce values of 6.0 (*n* = 10), 6.5 (*n* = 21), and 7.0 ng/mL (*n* = 5), which were similar to the EC_90_ calculated using our results. We also calculated the EC_50_ (5.33 ng/mL; 95% CI, 5.27–5.44 ng/mL) and EC_95_ (6.74 ng/mL; 95% CI, 6.28–6.93 ng/mL).

## Discussion

In this study, the EC_90_ of remifentanil was calculated as 6.48 ng/mL (95% CI, 5.96–6.83 ng/mL). This Ce is approximately 20% higher than that typically used in our hospital (5.5 ng/mL).

Previously, several studies have investigated the most effective method of alleviating hemodynamic responses to head fixation [3–7]. The authors introduced and compared various strategies, such as intravenous opioids, local anesthetic infiltration at the pin sites, a combination of intravenous fentanyl and local anesthetic infiltration, and scalp nerve blockade. However, no consensus exists to guide anesthesiologists in attenuating cardiovascular responses to head fixation. In our experience, intravenous fentanyl injection alone may not be sufficiently effective in many cases, and local anesthetic infiltration may not always be effective because sometimes the exact pin sites may not match the infiltrated scalp area. Furthermore, in some cases, surgeons reposition the skull pins after their first attempt at head fixation. We perform scalp nerve blockade as a routine practice in patients undergoing awake craniotomy. In our experience, the effect of scalp nerve blockade is usually highly potent. A previous study showed an approximately 6.8% increase in the MAP from the MAP_BL_ due to head fixation in patients who received scalp nerve block with bupivacaine [7]. In our study, the increase in MAP in patients with a remifentanil Ce of 6.5 ng/mL was approximately 12.3%. However, scalp nerve block is not always effective, and its performance requires extra time and training.

As mentioned previously, remifentanil has excellent characteristics and is commonly used, along with propofol, by anesthesiologists for TIVA with a TCI system during neurosurgery. If a remifentanil Ce that effectively reduces hemodynamic responses to head fixation is attained, the anesthesiologists can maintain a stable hemodynamic status with fewer drugs and use a simpler approach.

Recently, a similar study was conducted to determine the EC_50_ of remifentanil necessary to minimize the cardiovascular changes due to head fixation under TIVA with BIS monitoring [13]. The authors in that study used the Dixon up-and-down sequential allocation method. They found that the EC_50_ of remifentanil was 2.90 ng/mL (95% CI, 1.78–3.65 ng/mL) and showed that the EC_95_ was 4.28 ng/mL (95% CI, 3.85–4.41 ng/mL) via isotonic regression estimation. These values were lower than our results.

Several differences existed between their study and ours. First, we used the BCD method, but they used the Dixon up-and-down method (UDM). Second, while we defined the MAP_BL_ and HR_BL_ values as the levels observed during the steady state at 1–2 min before the head fixation, they defined the baseline values as the levels observed before induction of anesthesia. Third, we adjusted the propofol Ce based on the BIS in our study, but they fixed the propofol Ce during the trial.

We used the BCD method to determine the EC_90_ of remifentanil for alleviation of cardiovascular responses due to head fixation. Many preliminary studies have used the UDM of Dixon and Mood to determine the ED_50_/EC_50_. The UDM was designed to estimate the median threshold, including the ED_50_ or EC_50_ [11, 14]. Although it would be possible to estimate the ED_90_/EC_90_ or ED_95_/EC_95_ by extrapolation in a UDM study, extrapolation of a high-quantile effect dose/concentration from the tolerance distribution curve determined by the UDM is not adequate due to weak precision [11]. Therefore, the BCD method is more suitable for determining the high-quantile effect dose/concentration because a BCD study can be performed to target the EC_90_, permitting a direct estimation of the EC_90_ and avoiding unverifiable extrapolations from the EC_50_ value [11]. Although, the EC_95_ would be more attractive to clinicians, a previous study showed that a sample size of 40 resulted in significantly less precision than determination of the EC_90_ using a simulation test [15]. Therefore, we determined the EC_90_ using the BCD method with a sample size of about 40.

We used the MAP and HR values 1–2 min before head fixation as baseline hemodynamic values. In neurosurgery on the brain, acute arterial hypertension may induce intracranial hypertension in patients with intracranial tumors and peritumoral edema [16]. In addition, a previous study reported that intracranial pressure could be rapidly increased by head fixation in patients with a brain tumor [2]. So, we used these values as baseline values of MAP and HR because we thought it should be important to prevent the abrupt increase of hemodynamics due to head fixation.

In the present study, we aimed to maintain BIS values at between 40 and 50 during the operative period and at approximately early 40s, but ≥40, immediately before head fixation. BIS values between 40 and 60 are recommended as an adequate level for general anesthesia in several studies [17, 18]. Another recommendation states that the BIS should be greater than 45 because BIS values below 45 are associated with increased mortality [19–22]. Sessler et al. introduced the ‘Triple low’ concept (low minimum alveolar concentration (MAC), low MAP, and low BIS), which was a strong and highly statistically significant predictor of mortality [23]. However, the authors of the study interpreted the finding to indicate that high-risk patients were sensitive to anesthetics rather than as an indication that the ‘Triple low’ (low MAC, low MAP, and low BIS) was a causative factor of high mortality. Several previous studies have supported Sessler’s study [22, 24, 25]. However, some studies have also provided conflicting results on this issue [26, 27]. A recent study found that a “smart alarm” to indicate the ‘Double low’ (low MAP <75 mmHg and a low BIS <45) did not decrease mortality [28]. Moreover, we found that the 30-day, 90-day, 1-year, and 5-year mortality rates were 0/43, 1/43, 3/43, and 3/43, respectively, in our study. This result is comparable to previous studies, even with the small sample size of our study [22, 28, 29].

A previous study reported that a BIS value between 50 and 60 prior to tracheal intubation was inadequate to prevent an awareness reaction to tracheal intubation during propofol/alfentanil anesthesia, although no recall case was included in that study [30]. In addition, two case reports and one randomized controlled trial presented awareness reactions despite use of the recommended BIS level, 40–60 [31, 32]. Therefore, we maintained the BIS between 40 and 50 during whole operation, and a target BIS value near 40 (but ≥40) was used just before head fixation to avoid awareness.

No patients experienced bradycardia (HR < 45) in this study. However, four subjects experienced hypotension with an MAP of <55 mmHg and were administered ephedrine. The incidence of hypotension resulting from the use of a remifentanil Ce of 6.5 ng/mL in this study was 8.7% (2/23). Based on our experience, hypotension is not uncommon during neurosurgery using this anesthetic technique.

A limitation of our study was that the subjects did not represent the entire group of patients who required head fixation for craniotomy. Twenty-seven patients were excluded prior to consent due to a diagnosis of hypertension during the pre-assessment. These subjects were excluded because patients with chronic hypertension can show an exaggerated response to noxious stimulation [33]. Further research into safer use of remifentanil in a more heterogeneous patient population with an unstable hemodynamic status, such as hypertension, is needed. A combination of propofol and remifentanil anesthesia using TCI with local anesthetic injection at the pin site or scalp nerve blockade might be an appropriate strategy for patients with an unstable hemodynamic status. Remifentanil Ce values that are lower than that calculated in the present study (6.5 ng/mL) may be needed to maintain stable vital signs in patients receiving a combination of TIVA with local injection, scalp nerve block or any premedication; this should be verified in future studies. We determined that an EC_90_ of remifentanil of 6.5 ng/mL is necessary to alleviate hemodynamic instability caused by head fixation in a small sample of patients (*n* = 43) using the BCD method. However, this sample size is not sufficient to generalize our results to all patients requiring head fixation. Therefore, further studies are needed to validate our results.

## Conclusions

Adjustment of the remifentanil Ce to approximately 6.5 ng/mL before head fixation could prevent noxious cardiovascular responses in 90% of neurosurgical ASA I-II patients aged 20 to 65 years old during propofol TCI titrated to maintain BIS between 40 and 50.

## Abbreviations

BCD: biased coin design up-and-down sequential method
Ce: effect-site target concentration
EC: effective concentration
EC_90_: 90% effective concentration
ED: effective dose
HR: heart rate
HR_BL_: baseline heart rate
MAC: minimum alveolar concentration
MAP: mean arterial blood pressure
MAP_BL_: baseline mean arterial blood pressure
MIE: modified isotonic estimator
TCI: target-controlled infusion
TIVA: total intravenous anesthesia
UDM: up-and-down method
